# Scared but Powerful: Healthcare Chaplains’ Emotional Responses and Self-Care Modes during the SARS-Cov-19 Pandemic

**DOI:** 10.1177/1542305021993761

**Published:** 2021-04

**Authors:** Cate Michelle Desjardins, Anna Bovo, Mario Cagna, Martijn Steegen, Anne Vandenhoeck

**Affiliations:** Transforming Chaplaincy, Chicago, USA; Department of Medicine, Laboratory of Evidence Based Nursing, University of Padua, Padua,Italy; Diocese of Chiavari, Chiavari (GE), Italy; University Hospital Leuven, Chaplaincy Services, Leuven, Belgum; Faculty of Theology and Religious Studies, K.U. Leuven, Leuven, Belgium; Faculteit Theologie en Religiewetenschappen/Faculty of Theology and Religious Studies, K.U. Leuven, Leuven, Belgium

**Keywords:** Covid19, self-care, healthcare chaplaincy, emotion, spiritual care leadership, chaplain

## Abstract

Drawing from both the qualitative free-text responses and quantitative responses to an international survey of 1657 chaplains serving during the SARS-Cov-19 pandemic, we explore chaplains' emotional responses to the pandemic and how emotion connects to self-care. This paper reports on the modes of self-care practiced by chaplains, including modes reported as unavailable due to pandemic restrictions. Lastly, we explore how effective spiritual care leadership may mediate chaplain emotions and ultimately chaplain self-care.

## Introduction

Care of self, emotionally, physically, and spiritually, has long been an important aspect of spiritual caregiving and perhaps more so during the challenges to chaplaincy practices that have arisen during the SARS-Cov-19 Pandemic (White et al., 2019). Chaplaincy is a role with high emotional demand and high levels of emotional labor (Hochschild, 1983). A survey was distributed to chaplains through their professional organizations in the USA, Europe, and Australia including quantitative and open-ended questions on their experiences during the SARS-Cov-19 Pandemic between the second half of May and the beginning of June 2020. Responses from 1657 chaplains were collected. The responses evidenced mixed emotions around their role as chaplains and personal wellbeing during the pandemic. Permission to complete the survey and an ethics review were completed at the Ethics Committee at KU Leuven.

Responses were gathered from 36 countries with non-English responses included and translated into English. The data on self-care was analyzed by a team of researchers representing Belgium, Italy, and the USA. Further details on the survey and analysis methods are available in a separate paper (Snowden, 2021).

The survey primarily included close-ended questions, and some open-ended qualitative questions. Three quantitative questions assessed chaplain emotions and self-care during the Covid19 pandemic: how anxious were you at different time points during the pandemic? Which modes of self-care did you engage in? and Which modes were unavailable to you? No open-ended questions were asked regarding self-care, but multiple self-care themes appeared in the qualitative data. The open-ended answers were analyzed through thematic analysis. The authors went through the first 200-300 answers of each open-ended question, defined initial categories, then looked through rest of answers for new or contradictory themes. The authors divided questions due to the volume of data, and all categories were reviewed by other authors, continually discussed, and corrected or agreed to by consensus.

The themes discovered in the qualitative data were frequently related to chaplains’ role facilitating self-care practices for other healthcare providers (“staff care”) and not highly descriptive of chaplains’ own self-care practices. Chaplains wrote of the lack of time or means for appropriate self-care during the pandemic in their responses to open-ended questions.

In this paper we explore the emotional world of chaplains during the pandemic and discuss the results from quantitative questions related to self-care modes and practices. Lastly, we discuss the role of leadership in supporting appropriate chaplain self-care and describe some leadership themes in the data.

## Chaplains’ Mixed Emotions: Scared but Powerful

Mixed emotions have been deeply felt by chaplains during the pandemic in the contrasting feelings of positive and negative emotions, frequently at the same time. These feelings arise from the experience of being in isolation, distance from the normal activities of life, or in contact with hospitals and the day-to-day places of chaplaincy service.

The two emotional spheres most experienced by chaplains were “scared” but “powerful”: both of which profoundly influence self-care. There is a large sense of hopefulness in experiencing an optimistic state of mind, which stimulates creativity and faith. A participant wrote: *“spiritual life happens between the difficult and the impossible”*. Chaplains felt optimistic in being flexible and thinking in options instead of difficulties, and in being creative in their emotional support even without physical contact. They wanted to enhance skills to face trauma, anxiety, uncertainty. Walking through the pandemic challenge was like a powerful symbol of hope and friendship to deepen relationships and trust. A chaplain wrote: *“we are trouble-shooters”* who try to keep a clear line to give perspective and meaning in the long run.

Moving to the negative sphere of emotions, a helpless insecurity has been an intense and collective emotional state for many: *“I lost for two weeks the ability to care spiritually […] I was confused and had to re-orient myself”*. Other negative emotions were expressed as a *“profound feeling of moral guilty [sic]”*, loneliness and tiredness. Some were dealing with fear and uncertainty: *“we have become afraid of touching one another, or even standing”*; *“I’ve never felt this lonely before”*. There is also a level of anger, due to the frustration and powerlessness in engaging with hospitals and the feeling of being peripheral and not essential.

Besides this contrast of emotions as a mix of contradicting experiences lived worldwide, we can introduce the concept of labor. Emotional labor is defined as the effort made to transform heavy emotional moments in order to answer the feeling rules dictated within a specific environment (Hochschild, 1983). This is what has been experienced by many chaplains under pandemic. Emotional labor includes *“normalizing a variety of feelings and questions”* during this very abnormal time. *“I am acting all the time”* as there is a background fear. Chaplains practiced emotional labor by staying confident and calm, giving a sense of security and reassurance: the need to be *“calm in stormy waters”*. Other chaplains demonstrated the contradiction felt in comparison with others. *“Am I a hero or a victim?”* Chaplains felt confusion within themselves, seen as hero-chaplains while others were feeling victims caught in the system: *“should I be unafraid and step into patients’ situations or sit back and watch, feeling unable to help?”*.

### Living with Paradox

*“My role is to be present but, because of the pandemic, my role is to be absent to be present.”*

In this paradoxical sentence we can find a chaplain’s intuition that providing service is now hugely limited but that it’s also possible to explore and discover new ways to be effective as a chaplain. Many chaplains faced the experience of being obliged to keep physical distance, with a frustrating lack of non-verbal communication, and many times they may not even have been admitted into the workplace. It is objectively an impediment, but, for some chaplains, it became the chance of another way to provide effective service, mirroring the emotional world discussed above: scared but powerful. Many chaplains report problems using technology as a way to take care of patients and staff. Other chaplains report good outcomes, including having acceptance for what, due to the pandemic, is impossible to do and using technology for accessing people in a creative way.

It is possible to perceive in the chaplains’ writings fluctuating attitudes between different emotions, “mixed emotions” as per above. Below we have identified key paradoxes in the qualitative data that illustrate the mixed emotions of chaplains during the pandemic.

#### Absence – another way to be present

Chaplains gained during the pandemic: “*We were the only paramedical caregivers who were allowed to visit covid-19 patients. We lead moral deliberations. We visited all the departments of the hospital*”. Chaplains reported gaining a better visibility in the organization and a new acknowledgment of the importance of spiritual care they provide and the impact that their work has on residents/patients especially during difficult times: “*We could provide care where others don’t want to be or don't have enough time to stay*”.

At the same time, Chaplains experienced great losses during the pandemic: *“my employer obliged us to work remotely”*. From this decision comes a disappointment in the people that the chaplains were supposed to take care of: *“We lost touch with patients, families and staff. Some were angry or felt abandoned by us. We felt that we were not part of the team anymore and we were afraid that, after all the work we've done to be integrated, we will lose our place”.*

However, other chaplains similarly reported: *“my employer allowed me to work remotely”*: almost the same sentence of the previous “disappointment”. However, the evaluation is very different: *“I learned to be attentive to active listening. In the absence of body language and physical observations I can focus on the person's tone of voice, previous acquaintance, content, etc … I learned to increase the above skills to a heightened level”*.

Others were disappointed in their organization: *“My organization didn't embrace innovations to assure spiritual care”*. It seems that a lack of technological devices is the problem. But, again, a “swinging” evaluation from another chaplain that wrote: *“counseling using a phone call [or a video call] is less effective than presence, because we lacked all kinds of non-verbal communication”*. Still other chaplains discovered the core of their profession was not subject to changes in departments or to the needs of the pandemic: *“I found that the basic principles of chaplaincy are good: no matter the format of meeting. Just listen, listen, listen; nothing fancy is needed. People want to be heard and acknowledged”.*

Prayer was a deeply felt area of chaplaincy practice that shifted in the pandemic, and numerous chaplains experienced disappointment in these changes: *“Prayer at distance (using a call or video call) and distant active listening is without the benefits of body language mirroring (and supportive touch when appropriate)”*.

#### Presence, but physical distance using PPE

Some chaplains found that PPE empowered them to continue providing physical presence, and allowed them to care in a deeper way than before: providing “*emotional support, listening, religious care, etc, we had to drop the ‘fluffy’ language and… it worked well!”*.

Others were frustrated and upset with PPE usage, as illustrated by this quote: *“Wearing masks made it difficult to be understood by people with hearing problems or to understand people who speak softly or mumble: masks obscured faces and muffled voices. It was impossible holding a hand, rubbing an arm, sitting close, hugging a person who wanted that connection”*.

#### Presence with Staff

In the qualitative data, the use of the phrase “self-care” in over half of the instances was associated with chaplains facilitating self-care activities for staff, not with chaplains’ own self-care activities. Being present *“with staff became an opportunity for the team to self-reflect, to share fears and concerns, to consider the needs for self-compassion and pause in order to be ready to host patients, to offer bereavement support”*. Rituals, with staff and patients, often need body language and sometimes it was impossible to provide rituals even when physically present. One chaplain reported: *“Felt stuck behind a face mask when providing spiritual care on the unit”*.

### Four Emotional States Impacting Self-Care

In the exploration of chaplains’ emotional world described above, it is possible to further present and categorize four emotional states which are mirroring the four main qualitative questions asked in the survey. Those four questions were: What was lost during pandemic? What has changed? What was new? What was most effective in your new actions? It is interesting to notice the dual tension which seems to be always there within chaplains’ emotional world. Feeling lost while feeling belonging. Facing the unknown, while navigating the change.

#### Grief and feeling lost

The emotion of having lost something and being lost are both present. The main perceived loss relates to face-to-face conversation, touch, social activity. In the dimension of face-to-face interactions there is a profound sense of the loss of facial expressions (smile, mimics). Connection in the authenticity of warm and personal human encounters is missing and chaplains feel the void to emotionally engage and find their inner spontaneity and intensity in order to be present. Very few survey respondents had nothing to say around loss. However, many chaplains have been able to positively transform their reality: this leads us to the feeling of change.

#### What change feels like

The major change has been felt in the staff support. Chaplaincy has risen its image of not only being there for end-of-life rituals. There is much more awareness of its wider role by other professionals who also were in need of spiritual care in moments of crisis. The importance of spiritual care is starting to receive more recognition while everyone is asking questions like “*what is really important in my life?*”. This opens a dialogue in which chaplaincy plays a crucial role in connecting deeper with people, giving support in defining emotions and dealing with them. Creativity has been the main resource for change, embracing resilience and going beyond the feeling of loss. There is a sort of hope things will get back to this and the fear of chaplaincy forever being different: this leads us to the feeling of the unknown.

#### The unknown feeling

How long is it going to be like this? How long is possible to hold it together? Those questions drive again the profound inner debate between feeling helpless or paralyzed by the events versus feeling proactive and resilient in facing the unknown. It seems to be helpful in dealing with the unknown aspect of the pandemic talking about the change and being part of it as a pioneer. This leads to holding us together to achieve a feeling of belonging.

#### Feeling of belonging

“*It has been a wonderful worldwide grieving experience to cope within a big community*”. There is a common feeling of belonging with the worldwide loss of human physical connection, yet there is a desperate need for more intimate and authentic connections to nurture self-care: “*every human being is in need of it and people were happy to be reached out to, even through technology tools*”. There is a profound intimacy which happened through anticipated grief and bereavement. From this sense of being ‘in it together’ comes feelings of belonging that all are a part of this Pandemic world.

### Emotional Self-Care

Vulnerability is the main emotion identified in the survey response that leads to self-care: “*I need a hug, I need to give hugs, staff and patients need hugs and human touch*”. Chaplains have learned how to express their emotions through their eyes, trust their instinct and ask harder questions on the phone, ground themselves and work on their insecurity using daily writing: I learned to “*keep my own confidence, I was supporting myself*”. Caring for the self includes moving outside of their comfort zones with creativity, thinking outside the box. Sometimes it is *“hard to re-fill my energy during the day due to heavy conversations*” which take away “*hope, air and sense of humor”*. Thus, it is essential to help each other through this time: “*I heard of colleagues who were frozen and did not know how to adapt*”. There is a profound call for help to the institutions, asking them to answer and brainstorm novel solutions to the questions: how to adapt in changing times? How am I needed? What do I offer? Connection among chaplains is crucial and it is helpful through education and webinars to support each other in re-thinking how to be present. For some chaplains, finding someone who can touch them and care for them seems to “*do more spiritual good than one hour of praying with me*”. Emotional well-being is essential for self-care.

## Quantitative Self-Care Data: What Does It Tell us about Chaplains’ Self-Care Modes?

In the quantitative part of the survey, we asked three questions related to chaplain emotions and self-care: how anxious were you at different time points during the pandemic? Which modes of self-care did you engage in? and Which modes were unavailable to you? We did not ask for free-text, qualitative responses to these questions and thus do not have evidence to understand how people understood the nature of the questions, especially in the context of a multicultural survey.

Interpreting the results of the question “How anxious were you?”, it appears that chaplains keep calm as a general pattern, and report not being very anxious and not being afraid. However, these numbers are impacted by those who are not allowed to see patients and should we have only questioned those admitted into institutions then we would have had a better view of chaplains anxiety related to the virus. This question may have been interpreted by chaplains loosely, including anxiety about the virus, or about losing professional access (not being able to see patients) and loss of identity.

### Outer Resources

Regarding self-care modes specifically, the survey asked about engagement in both outer and inner self-care resources. Outer resources, those requiring others or a certain level of physical space (such as for “sport”, meaning exercise or collective sports) were less likely to be utilized as self-care modes by chaplains during the pandemic and more likely to be reported as “unavailable”. For example, sport was reported to be low overall by chaplains during the pandemic. This may have been a result of differences in use of language, with “sport” being a term much more common in the UK than the USA or Europe. However, when asked whether sport was unavailable, only 210 chaplains reported sport was unavailable to them – leading to a tentative conclusion that sport may not be the preferred mode of self-care by chaplains in non-pandemic times as well as pandemic.

Support in therapy, Supervision, and Intervision were modes of self-care reported to be utilizes only “sometimes” by many during the Pandemic. The authors also recognize that the term “Intervision” is primarily used among European chaplains to mean formal peer coaching, and is unknown amongst American chaplains. European chaplains represented only around 400 respondents in the survey, which may render results to the question about Intervision as a mode of self-care unreliable. Supervision was the most-reported “unavailable” mode of self-care during the Pandemic. One can imagine that during the height of the pandemic as leaders and chaplains were still learning all the ways to communicate with each other online, it may have taken a while before supervision was available online. This also raises questions about the perceived availability and effectiveness of leadership. Were leaders so overwhelmed with reported constant changes to chaplaincy practice and access that individual supervision and access was limited?

Chaplains frequently reported support from other chaplains, but not every day. Gathering was made harder amongst chaplains and many chaplains are alone in institutions, but chaplains still found ways to support each other.

### Inner Resources

Chaplains reported the most engagement in modes of self-care that were internal and in-line with the spirituality inherent in the chaplains’ profession: prayer, meditation, walking, silence, and speaking to friends and family. Only 13 chaplains reported that prayer was not available to them during the pandemic, and prayer and meditation were the highest reported self-care mode. Many chaplains also reported activities like walking to be a very frequently engaged activity, as well as speaking to friends and family. Considering that many in the general population lamented lost connection to friends and family during the pandemic, we hypothesize that perhaps chaplains, as they transitioned to the “many digital ways” of providing patient care, felt comfortable and connected enough through technology to still find distance-based relationships with friends and family nourishing. Chaplains identity is also rooted in caring, and this may have encouraged reaching out to friends and family, leading to a greater sense of connectedness and high reported relational self-care.

Engagement with hobbies was also quite high in the quantitative data. However, the researchers specifically suggested examples for this question of “reading and music”, both of which are hobbies available to continue in-home, which may have lead to high-reported engagement in hobbies.

Silence was a mode of self-care reported to be practiced “every day” more frequently than the categories “often” and “very often”. Silence may be the “base” for other inner-resources reported as practiced frequently: prayer and meditation. The number of respondents who did not answer self-care questions remained relatively stable for each mode, around 440, so we can conclude that all chaplains except around 100 implemented silence as a self-care strategy at least in part.

Time was reported as a highly utilized self-care practice, but also more frequently reported as “unavailable” during the pandemic. Sleep followed a similar pattern.

It is possible that while there was time available for self-care modes, when asked whether it was available chaplains were aware of the pressed nature of their work during the pandemic and sleep may have been impacted by the constant changes, even though chaplains did not report exceptionally high anxiety. The largest group said that they were getting sleep every day, but a large number said ‘Sometimes’. It may be that chaplains are so tired from hearing the stories, suffering, dealing with changes, that sleep is also a way out.

All the resources that we summed up, including inner and outer resources, were very much accessible to chaplains – with a few outer resources *less* accessible, and reported as less frequently practiced and engaged in.

## The Role of Spiritual Care Leadership in Chaplain Self-Care

Above we postulated that availability of leadership and supervision may have impacted chaplains’ ability to care for themselves effectively. Leadership is crucial in building high-quality and safe work environments, in stimulating creativity and innovation. The way in which leadership takes shape in a health institution has a huge impact on the resilience and wellbeing of caregivers. In the questionnaire chaplains mainly referred to leadership issues and the impact on their working attitude with regards to the following questions*:* ‘How prepared was your institution for the pandemic in the following months?’; ‘How clear was your role to you in the following months?’; ‘How do you think this pandemic has changed our profession?’; ‘Did your organization limit your access to covid-19 patients?’ and ‘Think about yourself, chaplains in general, and their place in the pandemic’. Four major themes recur throughout the answers: integration, preparation, communication and knowledge of the job content of chaplains. During the SARS Cov-19 pandemic chaplains experienced ‘good’ leadership in their institutions, but also examples of ‘bad’ leadership.

### Integration

Whether chaplains experienced good leadership in their institution or not during the first wave of the covid-19 pandemic had to do a lot the degree of integration to interdisciplinary and system management teams. Chaplains who have a seat at leadership tables had more chance to be fully involved in the care for covid-19 patients. Mostly, this is a product of ongoing advocacy over many years. One of the respondents suggests enlarging the understanding of ‘being present’ as chaplain. Being present as chaplain does not only mean being present with a patient, with their family, but also means being present at the leadership table and with staff. Well integrated chaplains experienced appreciation and had room for improvisation with deepening alliances with other care workers in the organization.

When there was a lack of leadership during the pandemic, chaplains experienced a lot of moral distress in the institution: “*Lots of people were left to make decisions on a personal level, which led to moral distress*”. Chaplains also report the negative impact on their self-confidence when management teams were terrified with the pandemic and took internal decisions that were in opposition to broader guidance which caused cognitive dissonance and confusion: “*the attitude of leadership and nursing seemed to be that everyone besides doctors and nurses would spread covid-19, so everyone besides doctors and nurses must be excluded from encountering covid-patients*”. On the part of the chaplain this attitude created angst and made the work seem meaningless.

### Preparation

The degree of integration often goes hand in hand with good preparation. Chaplains do mention that although every institution was rushed into the pandemic, the preparedness of leadership teams to unforeseen events was decisive in the way institutions called in chaplains. When leadership teams were prepared to a certain level there was enough mental space to be vigilant and to make proactive decisions: “*proactivity on part of pastoral care was valued by system leadership*”. Being well prepared to covid-19 on the level of the pastoral care team means that there are clear policies and guidelines for pastoral care to function safely. The low availability of personal protective equipment caused a lot of distrust: “… *but my confidence in medical leadership had dwindled … . Our hospital cut staff hours and therefore, paychecks, for all staff. Our department was cut 20%. Daytime dollars and cents? If I am to put out a fire, give me the proper tools*”.

### Communication

The way in which leadership teams communicated also impacted the resilience of chaplains. Transparent communication about decisions obviously helps to gear activities to one another. Chaplains mention that thorough and full response from organizational leadership helped to respond and prepare better pastoral care: “*leadership of every part in our institution met three times a week to coordinate what to do in which case*”. Relationships with management teams bumped into difficulties when they did not do well in keeping their staff informed. It could even engender suspicion about the sincerity of leadership teams: “*It appeared as if they wanted to keep all communication positive and upbeat*”. Chaplains also report the non-communication of cutting staff hours, paychecks. No communication about many schedule adjustments: “*we were expected to do more with fewer hours… Our hospital sailed banners about how they cared about the staff but did not show it to us directly*.”

### Knowledge of Chaplain Role

The results of the questionnaire show that the knowledge of what chaplains do is also decisive in which role was given to the chaplains during the first wave of the SARS-Cov-19 pandemic. One chaplain emphasized that it is important to name and claim the authority of the discipline that determines the responsibilities of a chaplain: “*chaplains were named an essential service, and we were allowed to go pretty much everywhere*”. Nevertheless, someone else mentions that he or she felt beleaguered by system leaders who did not understand spiritual care. He or she frequently felt inadequate. But there was also room for progression. Due to technical innovation chaplains who were considered non-essential at the start of the pandemic were able to fully integrate in the care for covid-19 patients: *“Some of us responded quickly, with innovative measures to continue to provide support via telephone. In this center, one day CPE was conducted in person. The next day everything was online via teleconference. We can innovate when we need to.”* (Note: CPE in this quote stands for Clinical Pastoral Education.)

At the very heart of self-care there is the relationship and connection to self. ‘Good leadership’ encourages caregivers to understand their needs to be their most constructive, effective and authentic self. Being well integrated in the organization, the ability to work proactively thanks to good preparations, transparent communication and clarity with regards to the job content of the chaplain are important markers with regards to how chaplains feel and function in the institution. It encourages chaplains to function at their best: “*I feel that I was able to provide some of the best ministry of my entire career as a chaplain. I continue to enjoy the deeper relationships with my colleagues in healthcare*”.

## Conclusion

The picture of how chaplains cared for themselves during the worldwide SARS-Cov-19 pandemic is complicated by the wide range of emotions chaplains experienced and the impact of leadership, or lack of leadership, on chaplain identity, role, and ability to practice self-care.

We expected in this paper to primarily describe modes of self-care as practiced by chaplains during the SARS-Cov-19 pandemic. While we have done so by describing the quantitative data and concluding that many chaplains turned towards inner modes of self-care while leaving behind or finding unavailable outer modes of self-care, we discovered in the qualitative data remarkable linkages between chaplains’ emotional responses to the pandemic, self-care, and leadership practices. If one mode of self-care is, as we surveyed in the quantitative data, receiving supervision, then the quality of and availability of leadership for chaplains during a pandemic is crucial to self-care. Further, chaplains turn to self-care out of their emotional responses to their work, which have been described by chaplains in the qualitative data as remarkably deep and also varied across personalities and workplaces. Chaplains appear deeply impacted in their identity and sense of “place” due to the SARS-Cov-19 pandemic, an emotional response that is also highly impacted by leadership, or “having a place at the table.” We suggest, then, that chaplain self-care arises out of and as a response to chaplain emotional responses, for which quality and quantity of leadership may be a mediating factor. Further research is necessary to explore this model of self-care further.

A limitation of this research is not having included free-text responses to the questions regarding self-care modes. Gathering more qualitative data about how chaplains were practicing, or not practicing, self-care directly would have strengthened this paper and helped us to determine how respondents interpreted the quantitative questions, especially in this cross-cultural survey. Further, questions going behind “what modes were unavailable” to rate what modes of self-care chaplains are practicing more or less during the pandemic would have strengthened our understanding of chaplain self-care modes. However, a strength of this paper is the wealth of data with rich emotional description by chaplains and the further breadth of the data across cultures, types of work sites, and years in the profession.

There is a need for further research in chaplain self-care practices globally, and further delineation of how different contexts, locations, and cultures impact chaplain self-care. Questions for further research include: how does leadership style impact chaplain self-care? How does the definition of a chaplain’s role and a chaplain’s sense of identity impact self-care? Are these inner modes of self-care inherent to chaplains only in a pandemic time or is this a pattern across the profession?

In conclusion, chaplains’ practices of self-care are a complex phenomenon that are impacted by far more than what modes of self-care are or are not available, drawing from chaplain emotional responses, and profoundly impacted by leadership.
